# Roles of medical education department: What are expectations of the faculty?

**DOI:** 10.12669/pjms.344.14609

**Published:** 2018

**Authors:** Saima Batool, Muhammad Ahsan Raza, Rehan Ahmed Khan

**Affiliations:** 1 Prof. Dr. Saima Batool, FCPS (Pediatrics), MHPE. Professor of Pediatrics, University College of Medicine, The University of Lahore, Lahore, Pakistan; 2 Dr. Muhammad Ahsan Raza, MPhil, CHPE. Associate Professor of Biochemistry, M. Islam Medical College, Gujranwala, Pakistan; 3 Prof. Dr. Rehan Ahmed Khan, FCPS, FRCS, MHPE, Ph.D. (Scholar). Professor of Surgery, Assistant Director Medical Education Department, Riphah International University, Islamabad, Pakistan

**Keywords:** Faculty, Medical education department, Perspective, Role

## Abstract

**Background & Objective:**

Pakistan like many Asian countries is investing in medical education to address increased societal needs and to meet the requirement of national and international accrediting bodies. Establishing medical education departments is part of this investment. The research question was “What are the expectations of faculty from medical education department?” The objective of this study was to explore the Faculty’s perception about the roles of medical education department and their suggestions for its future endeavors.

**Methods:**

A qualitative case study design was chosen for this study. Heterogeneous group of faculty members from basic and clinical sciences departments of University College of Medicine, Lahore were invited for this study. They represented a variety of disciplines, and seniority levels. They were queried about their perception of the roles of medical education department and were encouraged to give suggestions for better functioning of department. Data was collected by audio recording through focus group interviews. Data analysis was done using NVIVO 11 software.

**Results:**

Initially 55 nodes/codes emerged which were then condensed to 35 nodes. Out of these three main themes emerged. The three emergent themes were:

1. Knowledge about the roles of medical education department.
2. Interactions with the medical education department.
3. Future Prospects of the medical education department.

Roles of medical education department identified by the faculty were mainly related to faculty development, curriculum planning and implementation, student support, policy making for student induction, improving teaching strategies, student assessment, quality assurance and accreditation of the medical college. Faculty development not only encompassed faculty training but also provision of opportunities for research and curriculum development. Student support was found to be a neglected role and faculty members suggested it to be an important area to be looked upon by medical education departments.

**Conclusion:**

Institutions must ensure consultation with faculty members and should take proactive measures to sustain change, including giving ownership and team building among the faculty members.

## INTRODUCTION

In Pakistan, first Medical education department was established by the College of Physicians and Surgeons, Pakistan (CPSP) in 1979, with the objective to support the college in the development of Postgraduate training programs and examination system, as well as, to help training the faculties for these programs. In 2008 Pakistan Medical and Dental Council (PMDC) sent a directive to all Public and Private medical colleges in the country to set up Departments of Medical Education (DME).

Setting up a department of medical education can be seen as a major change in a medical school in response to various pressures, expectations and changes in society, education and medicine.^1^

Benor (2000) predicted that, in the future, society is likely to impose a requirement for certification of medical teachers.^2^ Taking the development of DME as a change, faculty members’ perceptions and expectations in this regard need to be understood in order to sustain this change.^3^

A major function of medical education department is to help prepare the teaching staff with the necessary skills to undertake effectively their roles as medical teachers.^4^ Other well-known functions are curriculum development, service provision, assessment and ensuring the accreditation of medical college.^5^ The aim of this study was to explore faculty’s perspective about the roles of medical education department.

## METHODS

An exploratory case study approach was employed to identify the emerging concepts and relationships. A central question for the study was put forward and during the interviews, few more questions were asked.^6^ Selective, axial and open coding techniques were applied.^7^

The Constructivist ontology approach used helped in providing a wide range of opinions from the participants.

Constructivists have a world view (ontology) or a manner of understanding their own and others’ views about the nature of reality that is based on a belief that “human beings are active participants in the researched world” and that “social phenomena are more than the sum of their parts and can be understood only holistically”^7^. The epistemological basis of this study is the concept of `Constructivism`. The concept of constructivism as described by Creswell, came from Mannheim and from works such as Berger and Luekmann’s in 1967 and the Social Construction of Reality by Lincoln and Guba’s Naturalistic Inquiry in 1985 8.

The questions asked from the focus group participants were:

1. What are your perceptions about roles of medical education department?
2. What are the good and bad experiences while working with medical education department?
3. How the medical education department should function in future?

The study was conducted in University College of Medicine, the University of Lahore, between January to August 2016. The study population was faculty members, who were selected on the basis of their teaching experience. A purposive sampling technique was chosen and a total of sixteen faculty members were identified, four each, in four focus group interviews.

The data was collected through focus group interviews, audio recorded with two mobile phones and transcribed simultaneously, to avoid missing any information. A semi-structured interview approach was used and a uniform set of open-ended questions were asked to each focus group.^8^ The questions were put forward according to the knowledge gap identified through the literature search. The assistant moderator also took hand written notes to ensure transparency. The transcriptions were sent back to the interviewees for member checking.^8^

A thematic analysis of the data was completed using NVIVO 11 software and by manual analysis. Initially open coding generated nodes/codes, which were used to define themes. Axial codes were used to define correlation between themes. Merger of open and axial codes helped in making subthemes.

The credibility (internal validity) and conformability of the results was achieved by thick description, member checking and reflexivity.^9^ The transcripts were sent back to the participants for member checking and they were allowed to edit their responses.^10^ Inter-interviewee comparison and rich, thick description were two other methods employed in this context.^11^,^12^

## RESULTS

Thematic analysis identified three themes/categories. The emergent themes were:

1. Knowledge of faculty about the roles of medical education department.
2. Interactions with the medical education department.
3. Future Prospects of the medical education department.

**Fig.1:**
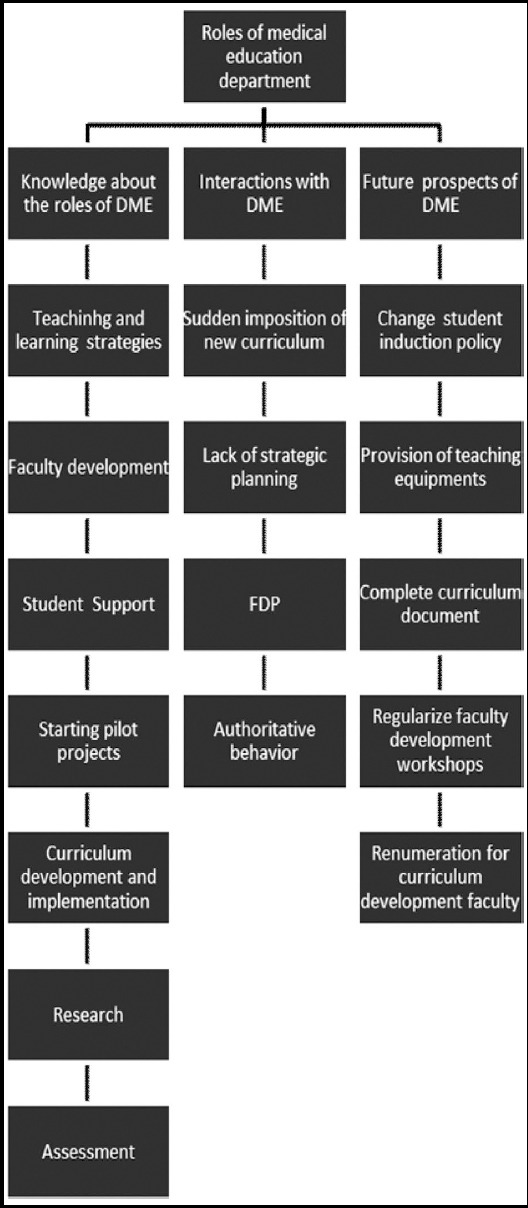
Hierarchy of themes and subthemes.

The hierarchy of themes and their relationships are shown in Fig.1. The participants were of the view that medical education department has far more scope than what it is doing currently. Besides well-known roles of faculty development, student support, research and curriculum development,^5^ policy making for student induction, provision of logistics and equipment and maintaining student portfolios were the new roles identified by the faculty. As participant A said “They should conduct aptitude test and interviews not just the merit alone and while devising this test should involve the concerned departments as well”.

Most of the respondents, especially the junior faculty members were dissatisfied with the current functioning of the DME. They thought that the concepts and terminologies that the medical educationists use are confusing for them. While the senior faculty noted lack of persuasion, poor strategic planning and abrupt implementation of its policies to be the worth mentioning deficiencies. Participant D talked about medical education concepts being confusing for her: “After attending so many programs by DME, I feel that these words like spiral curriculum, integration etc. are very new for me. I am still confused about medical Education”.

The participants were asked about their suggestions for better functioning of the department. Many of them believed that a systematic approach to identify the strengths and weaknesses of DME through faculty’s feedback would help plan its future endeavors.

## DISCUSSION

### Theme-1: Knowledge about the functions of medical education department

The department of medical education has a wide-ranging function that includes research, teaching, service provision including advice and support in curriculum development and assessment. Another well-known role is fostering the careers of the academic staff.^5^

Twelve out of 16 members (75%) identified Faculty development workshops to be an effective learning experience. They categorically identified faculty development as the most important responsibility of medical education department.^13^

Faculty perceives that the DME is ‘taking over the curriculum’ and that is threatening for them and in turn they may respond by ‘attacking’ the DME.^13^ Similar findings have been identified by authors, as the participants find the DME ‘a sudden change’, which was being imposed on them and medical educationists use ‘difficult words’ which are confusing for them.

Provision of teaching rooms, IT facilities and other logistic support is the responsibility of the medical college’s management.^14^ Surprisingly, the authors have found that the faculty looks forward to DME for this purpose. It seems to be an undue expectation from a department mainly concerned with educational and research activities.

### Theme-2: Interactions with the medical education department

The junior staff members are more enthusiastic about faculty development workshops as compared to senior ones. Similar findings have been found by other researchers.^14^

Unexpectedly no participant in this study talked about teaching and assessing professionalism at undergraduate level. Probably this is because of lack of awareness among faculty that Professionalism must be taught explicitly in the formal curriculum.^15^

Remarks about unpleasant interactions are related to DME being a ‘sudden change’ for the faculty. Castillo (2014) claims that “Acceptance of change in an educational institute can only be achieved if the expectations of those involved in the change process are sought for and given weightage at every step of change management”.^16^ That is why; the outcomes of change must meet the expectations of the participants.

### Theme-3: Future Prospects of medical education department

Improvement in the faculty development activities is the most frequentlyrecommended activity by the interviewees. Inviting external speakers, increasing the frequency of activities to fortnightly and giving time and duty relaxations to those attending should are ensured by DME. These findings are similar to other studies.^17^ Curriculum mapping, introduction of smart phone learning and conducting pilot projects with the faculty are other tasks identified by the participants.^18^,^19^

A ‘new task’, the faculty has identified is provision of teaching space, logistics and equipment to the faculty. The authors haves not been able to find any other study which states this to be a function of DME, rather the researchers have clearly stated it to be responsibility of management of medical school.^13^

### Limitations of the study

Faculty members from only one medical college were included in this study so further research is warranted on this topic on a larger scale. Secondly, as only 5 out of 16 participants in this study had attended a formal program or are enrolled in master of Medical education (MME), the results can differ if the study is repeated on a sample of participants who have received formal training in medical education. Purposive sampling and a small sample size, used have imposed restrictions regarding any attempt to generalize these results.

Researchers can study other aspects like seeking Student’s or the management’s perspectives or Performing longitudinal study to see the effects of reforms in light of recommendations of this study.

### Recommendations

The findings of this study point to following recommendations for addressing and improving the functioning of medical education department. DME should:

1. Procure the support of the dean and other powerful advocates within the medical school.
2. Ensure a non-threatening and non-judgmental approach to the school and faculty members.
3. Employ appropriate staff for itself with an experience in developing integrated curriculum.
4. Convince the management to provide funding for a few years until self-supporting.
5. Establish contact with other groups/DME both nationally and internationally.

## CONCLUSION

Medical Education is a specialized field that is becoming a necessity for any medical school in the World. In a qualitative case study the faculty members highlighted various roles that a medical education department is expected to perform. Most of the faculty members were dissatisfied with the department’s current functioning. The study concludes that the institution must ensure consultations with faculty members and should take proactive measures to sustain change including giving ownership and team building among the faculty members. As Dent (2017) recommended, that proactive measures against resistance or non-acceptance of an institutional change are better than addressing the resistance once it had happened, because it could be viewed as being reactionary.^14^

### Authors’ Contribution

**SB** conceived, designed and facilitated focus group discussion session. Final editing of manuscript was also done by her.

**MAR**, was the assistant facilitator in Focus Group discussion and manuscript writing.

**RAK** helped in editing of the manuscript and critically reviewed it as an expert Medical educationist.

**SB** takes the responsibility and is accountable for all aspects of the work in ensuring that questions related to the accuracy or integrity of any part of the work are appropriately investigated and resolved.
